# Analysis of Ale

**Published:** 1875-12

**Authors:** 


Why Some People are poor.—Cream is allowed to mould and spoil. Silver spoons are used to scrape kettles. The scrubbing-brush is left in the water. White-handled knives are thrown into hot water. Brooms are never hung up, and are soon spoiled. Dish-cloths are hung up where mice can destroy. Tubs are left in the sun to dry and fall apart. Clothes are left on the line to whip to pieces in the wind. The pie-crust is allowed to sour instead of making a few cakes for tea. Dried fruit is not taken care of in season, and becomes wormy. Vegetables are thrown away that would do to warm for breakfast. The cork is left out of the sugar jar, and flies take possession. Bits of meat are thrown out that would make minced meat or hash. Coffee, tea, pepper, and spices, are left to stand open and lose their strength. Pork spoils for the want of salt, and beef because the brine wants scalding.

